# Approach to Peripheral Neuropathies Leading to Hospital Admission

**DOI:** 10.1212/NE9.0000000000200341

**Published:** 2026-07-31

**Authors:** Reece M. Hass, Ashley R. Santilli, Jennifer M. Martinez-Thompson, Marcus V. Pinto

**Affiliations:** From the Department of Neurology, Mayo Clinic, Rochester, MN.

Peripheral neuropathies present with varying degrees of sensory, motor, and autonomic symptoms, or neuropathic pain.^1^ Peripheral neuropathies may prompt hospitalization, often because of the degree of disability or rapid progression relative to neuropathies encountered in the outpatient setting. We propose a stepwise approach to diagnosis in this patient population (Figure).

**Figure:**
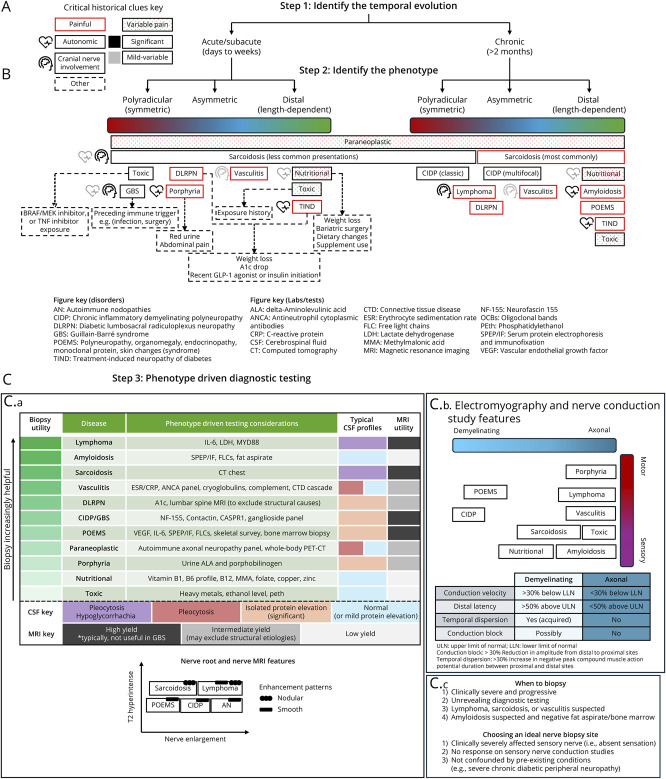
A Stepwise Approach to Hospital Peripheral Neuropathies Evaluation starts by identifying the temporal evolution (A. Step 1). Typical phenotypic presentations of peripheral nerve disorders, identified through neuromuscular examination and screening for relevant historical clues, are shown in B. Step 2. From here, an appropriately tailored diagnostic work-up based on suspected etiology can be conducted (Step 3), typically starting with differential driven laboratory testing (C.a Step 3). Electrodiagnostic testing (C.b Step 3) is most useful in identifying suspected demyelination, extending pattern assessment when clinically uncertain, and in choosing nerve biopsy targets (C.c Step 3).

Evaluation begins with identifying the temporal evolution and characterizing the clinical phenotype. Diagnostic testing is guided by these features and should generally be approached from minimally invasive to more invasive testing as clinically appropriate.
